# SOCIAL FUNCTIONING AS AN IMPORTANT CLINICAL TARGET DURING CARE TRANSITIONS FROM SKILLED NURSING FACILITIES

**DOI:** 10.1093/geroni/igz038.573

**Published:** 2019-11-08

**Authors:** Kelsey Simons, Whitney L Mills, Emily S Bower, Suzanne Gillespie, Kimberly Van Orden

**Affiliations:** 1 VISN 2 Center of Excellence for Suicide Prevention, Canandaigua, New York, United States; 2 Center for Innovation in Long-Term Services and Supports, Providence VA Medical Center, Providence, Rhode Island, United States; 3 Canandaigua VA Medical Center, Canandaigua, New York, United States; 4 University of Rochester Medical Center, Rochester, New York, United States

## Abstract

Care transitions from skilled nursing facilities (SNF) to home signify a period of medical risk for older adults. They also present opportunities for clinical interventions to reduce these risks and to enhance or maintain patients’ quality of life. A substantial body of research has been published on improving late life care transitions (CTs), including the development of standardized CT models for acute care. However, such models typically focus on improved coordination of medical services; overlook the need to address psychosocial well-being and social connectedness; and have rarely been implemented in SNFs. This poster will present a conceptual model of social functioning in older adults that draws on constructs from the World Health Organization’s World Report on Ageing and Health (2015). We propose that social functioning is a key part of overall functioning among older adults who use SNFs and is simultaneously influenced by physical, psychological, and cognitive functioning. To illustrate our model, we will present results of a qualitative study (n= 21) that describes declines in social functioning following care transitions to the community from VA SNFs. Implications for clinical practice include the need to better integrate social functioning in clinical assessments, goal setting, discharge planning, and coordination of care activities. The need for additional research on this topic will also be addressed. Our project is highly relevant to the overall conference theme “Harnessing the Power of Networks” as it presents a conceptual model and study findings related to social connectedness and social functioning in older adults who use SNFs.

